# DNA methylation status of *NKX2-5, GATA4* and *HAND1* in patients with tetralogy of fallot

**DOI:** 10.1186/1755-8794-6-46

**Published:** 2013-11-01

**Authors:** Wei Sheng, Yanyan Qian, Huijun Wang, Xiaojing Ma, Ping Zhang, Lianwei Diao, Quan An, Long Chen, Duan Ma, Guoying Huang

**Affiliations:** 1 Children Hospital of Fudan University, Shanghai 201102, China; 2 Key Laboratory of Molecular Medicine, Ministry of Education, Department of Biochemistry and Molecular Biology, Institutes of Biomedical Sciences, Shanghai Medical College, Fudan University, Shanghai 20003, China; 3 Shanghai Key Laboratory of Prevention and Intervention of Birth Defects, Shanghai 201102, China; 4 Department of Forensic Medicine, Fudan University, Shanghai 20003, China; 5 Wuhe Chinese Medicine Hospital, Wuhe 233300, China

**Keywords:** DNA methylation, NKX2-5, *GATA4* and *HAND1* genes, Tetralogy of fallot

## Abstract

**Background:**

*NKX2-5*, *GATA4* and *HAND1* are essential for heart development, however, little is known regarding their epigenetic regulation in the pathogenesis of tetralogy of fallot (TOF).

**Methods:**

Methylation levels were measured in three regions of *NKX2-5* (M1: -1596 bp ~ -1374 bp, M2: -159 bp ~ 217 bp and M3: 1058 bp ~ 1524 bp), one region of *GATA4* (M: -392 bp ~ 107 bp) and three regions of *HAND1* (M1: -887 bp ~ -414 bp, M2: -436 bp ~ 2 bp and M3: 37 bp ~ 398 bp) using the Sequenom MassARRAY platform. QRT-PCR was used to analyze *NKX2-5* and *HAND1* mRNA levels in the right ventricular myocardium of TOF patients.

**Results:**

TOF patients had a significantly higher *NKX2-5*_M3 median methylation level than controls (41.65% vs. 22.18%; *p* = 0.0074; interquartile range [IQR]: 30.46%–53.35%, N = 30 and 20.07%–24.31%, N = 5; respectively). The *HAND1*_M1 median methylation level was also significantly higher in TOF patients than controls (30.05% vs. 17.54%; *p* = 0.0054; IQR: 20.77%–40.89%, N = 30 and IQR: 14.69%–20.64%; N = 6; respectively). The methylation statuses of *NKX2-5*_M1, *NKX2-5*_M2, *GATA4*_M, *HAND1*_M2 or *HAND1*_M3 were not significantly different in TOF patients compared to controls. The methylation values for *NKX2-5*_M3 were negatively correlated with mRNA levels (r = - 0.463, *p* = 0.010, N = 30) and there was a significant association between *HAND1*_M1 methylation status and mRNA levels (r = - 0.524, *p* = 0.003, N = 30) in TOF patients.

**Conclusions:**

Aberrant methylation statuses of the *NKX2-5* gene body and *HAND1* promoter regions are associated with the regulation of gene transcription in TOF patients and may play an important role in the pathogenesis of TOF.

## Background

Tetralogy of fallot (TOF) is a cyanotic, congenital cardiac defect that is caused by improper development of the right side of the heart [1]. TOF occurs in 3.6 of every 10,000 live births and accounts for 10% of all congenital heart defects (CHD) [2]. TOF is a complex heart condition that is characterized by a malalignment of the conal septum, which leads to a rightward deviation of the aorta and results in a large ventricular septal defect, along with varying degrees of right ventricular outflow tract narrowing [3]. Although treatment has advanced dramatically over the past few decades, the exact etiology of TOF is unknown. There are still some TOF patients (0.5% to 6%) that suffer sudden cardiac death, despite undergoing treatment [4]. Genetic studies have identified numerous genes that are responsible for inherited and sporadic congenital heart diseases. Most of these, including *NKX2-5, GATA4* and *HAND1*, encode transcription factors that regulate specific phases of heart development [5]. Mutations in *NKX2-5*, the gene that regulates cardiac development, have been identified in patients with TOF [6,7]. *GATA4* is thought to regulate cardiac gene expression and physically interacts with *NKX2-5*. Mutations in *GATA4* may lead to defective interactions with *NKX2-5*, leading to congenital heart diseases [8]. *HAND1* is a basic, helix-loop-helix transcription factor that is essential for mammalian heart development. Mutations in this gene have been reported in patients with ventricular septal defect (VSD) [9,10]. Mutations of single genes, including *JAG1*[11,12], *JAG5*[13], *ZFPM2/FOG2*[14] and *TBX20*[15], have reportedly been found in TOF patients. However, single gene mutations or absences have only been found in a small percentage of patients and are not considered to be the main cause of TOF [16]. Furthermore, reports of somatic gene mutations in formalin-fixed, paraffin embedded cardiac tissue have not been confirmed in subsequent studies with fresh, frozen cardiac tissue, which consistently demonstrate an absence of somatic gene mutations. In fixed cardiac tissue, formalin can cause random base damage, affecting polymerase chain reaction (PCR) fidelity [17], thereby increasing the risk for poor data quality. Therefore, somatic mutation data obtained in fresh, frozen cardiac tissue may be more accurate and the incidence of single gene mutations is likely lower than is indicated from studies of fixed cardiac tissue. Interestingly, most of the genes above have shown significant changes in expression in the cardiovascular tissue of TOF patients [18]. Epidemiological data has also indicated that environmental influences may play a role in the etiology of TOF [19]. Epigenetic influences must be considered in order to understand the mechanisms that lead gene-environment interactions to cause disease [20]. Currently, the most widely studied epigenetic modification in humans is DNA methylation, which occurs almost exclusively in the context of CpG dinucleotides that control the transcriptional activity of genes [21]. Aberrant DNA methylation can result in silencing gene expression and functional inactivation [22]. This has been demonstrated in various human diseases, such as glioblastoma [23], chronic lymphocytic leukemia (CLL) [24] and invasive cervical cancer (ICC) [25]. Although mutations in *NKX2-5, GATA4* and *HAND1* have been found in patients with TOF, little is known about changes in these genes due to DNA methylation.

The goal of the present study was to explore DNA methylation changes in *NKX2-5, GATA4* and *HAND1* and to examine the epigenetic regulation of these genes in the right ventricular myocardium of TOF patients. These results may offer a deeper understanding of the etiology of this disease and provide important clues for the development of new treatments for TOF.

## Methods

### Patients and controls

TOF cases were obtained from the Children’s Hospital of Fudan University, Shanghai, China. Cardiovascular diagnosis was done using echocardiography. All TOF subjects were assessed for 22q11.2 deletions; only TOF patients without the 22q11 deletion syndrome were included. A total of 30 patients with TOF were studied, including 20 (66.7%) males and 10 (33.3%) females, ranging in age from 0.25 to 4.0 years (mean ± SD: 1.13 ± 0.85 years).

The control group was comprised of autopsy specimens from normal subjects that had died as a result of accidents. Specimens were collected at the Forensic Medicine Department of Fudan University, Shanghai, China. Control samples were chosen in which the time interval between death and autopsy was as short as possible so that any delays before autopsy would be taken into account. The post mortem interval (PMI) for the control samples was no more than 24 hours. Specimens from six age-matched normal controls were obtained, including 4 (66.7%) males and 2 (33.3%) females, ranging in age from 0.5 to 4.5 years (mean ± SD: 1.73 ± 1.44 years). Characteristics of the study subjects are summarized in Additional file 1: Table S1.

All tissue samples were obtained from the right ventricular myocardium tissues immediately after surgical resection or autopsy and stored in RNAlater^®^ (AMBION, Inc., Austin, TX, U.S.) until use to exclude any tissue heterogeneity that may affect methylation results.

This study was approved by the local ethics committee of Fudan University. Written informed consent was obtained from the parents or relatives of all study subjects.

### DNA extraction and sodium bisulfite conversion

A QIA amp DNA Mini Kit (Qiagen, Hilden, Germany) was used to extract genomic DNA, according to the manufacturer’s instructions, from the heart tissue samples of TOF patients and controls. The concentration and purity of genomic DNA were measured via absorbance at 260 and 280 nm using a NanoDrop^TM^ 1000 Spectrophotometer (Thermo Scientific, Wilmington, U.S.). Sodium bisulfite modification of genomic DNA was done, strictly according to manufacturer’s instructions, using an EZ DNA Methylation Kit™ (Zymo Research, Orange, CA, U.S.). Sequencing results confirmed that more than 99.0% of cytosine residues were converted. The bisulfite converted DNA was re-suspended in 10 μl elution buffer and stored at -80°C until the samples were ready for analysis.

### Quantitative MassARRAY analysis of gene methylation status

Quantitative methylation analysis for *NKX2-5*, *GATA4* and *HAND1* was performed based on base-specific cleavage and MALDI-TOF mass spectrometry, as recommended by the manufacturer, using the MassArray EpiTyper (Sequenom, San Diego, CA, U.S.). The Sequenom groups have demonstrated the robustness of this approach for quantifying methylated and unmethylated DNA [26]. The primers used in this study were designed using http://epidesigner.com (Additional file 1: Table S2.). For each reverse primer, an additional T7 promoter tag was added for *in vivo* transcription and a 10-mer tag was added to the forward primer to adjust for the melting temperature differences. All experiments were performed as described previously [27].

The accuracy of the methylation analysis results from the MALDI-TOF MassARRAY (Sequenom) were confirmed by pyrosequencing and bisulfite sequencing PCR (BSP) [28].

### RNA extraction and quantitative RT-PCR

Total RNA was extracted from heart tissue samples using Trizol Reagent (Invitrogen, CA, U.S.), according to the manufacturer's instructions. RNA was reverse-transcribed using a PrimeScript RT reagent Kit with gDNA Eraser (Perfect Real Time, TaKaRa, Otsu, Shiga, Japan) and the integrity of synthesized cDNA was confirmed using glyceraldehyde 3-phosphate dehydrogenase (*GAPDH*) as the endogenous control. Quantitative RT-PCR was performed in a 7900 real-time PCR system using SYBR Premix Ex Taq GC (Perfect Real Time, TaKaRa) using a 10 μl reaction volume, containing 5 μl SYBR Premix Ex Taq GC, 0.2 μM of each primer, 0.2 μ ROX1 Reference Dye and 100 ng cDNA. Reactions were performed in triplicate and analyzed using an ABI 7900 Sequence Detection System (Applied Biosystems). Relative expression levels were calculated according to the standard 2^-ΔΔCt^ method using beta-2 microglobulin (*B2M*) and the *GAPDH* gene as the endogenous control for normalization. Primer sequences used in the QRT-PCR analysis are listed in Additional file 1: Table S3.

### Statistical analysis

Data were analyzed using GraphPad Prism (version 5.0; GraphPad Software Inc., San Diego, CA, U.S.) and SPSS (version 13.0; SPSS Inc., Chicago, IL, U.S.). The Mann–Whitney test was performed to evaluate the significance of any differences between the TOF and control groups. Spearman correlation analysis was performed to evaluate the correlations between the methylation statuses and mRNA levels of the *NKX2-5* and *HAND1* genes. All statistical analysis was 2-sided and *P* < 0.05 was considered statistically significant.

## Results

### Methylation levels for *NKX2-5, GATA4* and *HAND1* genes in TOF patients and controls

**Figure 1:**
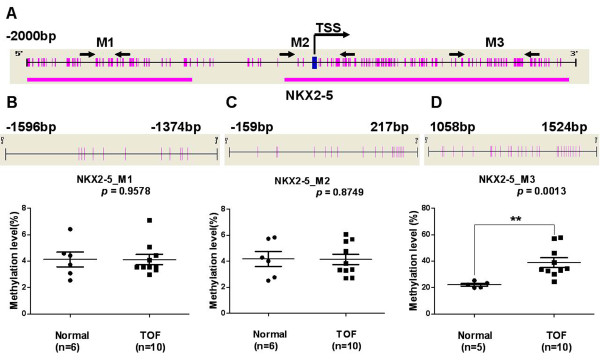
**Median methylation levels for *NKX2-5 *in TOF patients and controls. (A)** The schematic represents the distribution of the CpG site and CpG island in the *NKX2-5* gene; **(B)***NKX2-5*_M1 median methylation level; **(C)***NKX2-5*_M2 median methylation level; **(D)***NKX2-5*_M3 median methylation level. All of the values represent the median with the interquartile range. TSS, Transcription Start Sites; red vertical line, CpG sites; red thick bars, CpG islands; M,MassARRAY amplicon; The region between arrows, target amplicon. *p < 0.05, **p < 0.01, ***p < 0.001 (Mann–Whitney test).

**Figure 2:**
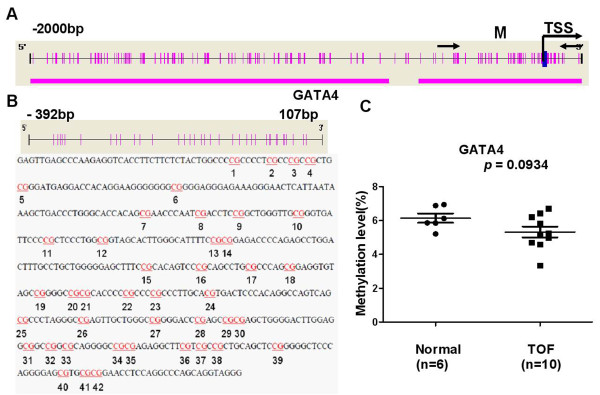
**Median methylation levels for *GATA4 *in TOF patients and controls. (A)** The schematic represents the distribution of the CpG site and CpG island in the *GATA4* gene; **(B)** The sequence shown represents a 500 base pair fragment (positions -392 – 107) in the 5′-UTR of *GATA4*; **(C)***GATA4*_M median methylation level. All of the values represent the median with the interquartile range. TSS, Transcription Start Sites; red vertical line, CpG sites; red thick bars, CpG islands; M,MassARRAY amplicon; The region between arrows, target amplicon. *p < 0.05, **p < 0.01, ***p < 0.001 (Mann–Whitney test).

**Figure 3:**
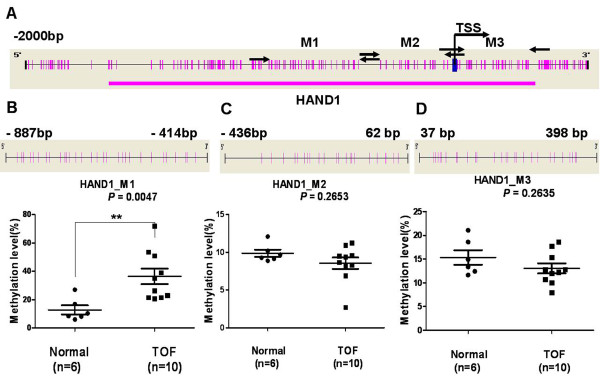
**Median methylation levels for *HAND1 *in TOF patients and controls. (A)** The schematic represents the distribution of the CpG site and CpG island in the *HAND1* gene; **(B)***HAND1*_M1 median methylation level; **(C)***HAND1*_M2 median methylation level; **(D)***HAND1*_M3 median methylation level. All of the values represent the median with the interquartile range. TSS, Transcription Start Sites; red vertical line, CpG sites; red thick bars, CpG islands; M,MassARRAY amplicon; The region between arrow, target amplicon. *p < 0.05, **p < 0.01, ***p < 0.001 (Mann–Whitney test).

The methylation statuses of *NKX2-5, GATA4* and *HAND1* genes were analyzed using the Sequenom MassARRAY platform. Three amplicons, including three regions, in *NKX2-5* (M1: -1596 bp ~ -1374 bp, M2: -159 bp ~ 217 bp and M3: 1058 bp ~ 1524 bp, Figure 1A), one amplicon, including one region, in *GATA4* (M: -392 bp ~ 107 bp, Figure 2A) and three amplicons, including three regions, in *HAND1* (M1: -887 bp ~ -414 bp, M2: -436 bp ~ 62 bp and M3: 37 bp ~ 398 bp, Figure 3A) were analyzed in specimens from 10 patients with TOF and 6 age matched controls. Prior to analysis, strict quality control was performed to remove potentially unreliable measurements. The CpG units that failed to produce data from more than 30% of samples (unreliable CpG units) and samples missing more than 30% of the data points (unreliable samples) were discarded [29].

The methylation level of the *NKX2-5*_M3 amplicon was significantly higher, with a median of 34.12% (IQR: 32.03%–48.86%, N =10) in TOF patients, compared to a median of 22.18% in controls (IQR: 20.07%–24.31%; *p* = 0.0013, N = 5, one male, aged 1.9 years was not included, Figure 1D). Methylation levels in the *NKX2-5*_M1 and *NKX2-5*_M2 amplicons were not significantly different between the TOF and control subjects (Figure 1B and Figure 1C).

There was no significant difference in the methylation level for *GATA4* _M when comparing the TOF and control subjects (Figure 2C). The methylation level of *HAND1*_M1 was significantly higher in TOF subjects, with a median of 30.77% (IQR: 21.59%–51.65%, N = 10) compared to 17.54% (IQR: 14.69%–20.64%; *p* = 0.0047, N = 6, Figure 3B) in the control group. No significant difference was observed in the methylation levels for *HAND1*_M2 and *HAND1*_M3 when comparing TOF and control subjects (Figure 3C and Figure 3D, respectively).

**Figure 4:**
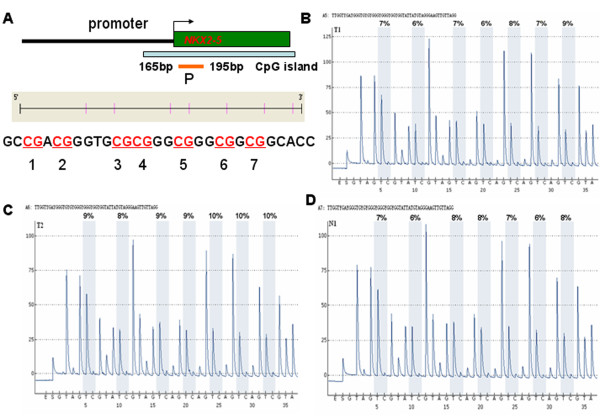
**Pyrosequencing results for *NKX2-5*. (A)** A schematic of the genomic structure of the *N*KX2-5 gene. The region analyzed for DNA methylation is depicted by the orange bars with the numbers and sequences showing the location of pyrosequence production with respect to TSS; **(B)** T1 sample pyrosequencing results; **(C)** T2 sample pyrosequencing results; **(D)** N1 sample pyrosequencing results; T, TOF samples; N, normal samples; TSS, Transcription Start Sites.

**Figure 5:**
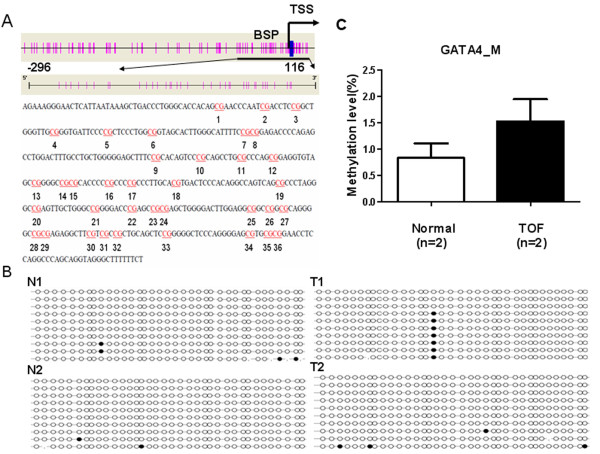
**Bisulfite sequencing PCR (BSP) results for *GATA4*. (A)** A schematic represents the distribution of the CpG site in the *GATA4* gene and the analyzed sequence represents a 413 base pair fragment (positions -296–116) in the promoter region of GATA4 gene; **(B)** BSP sequencing results, N1 and N2 represent the normal control, T1 and T2 represent the TOF subjects; **(C)** Methylation level for GATA4_M.

Pyrosequence was used to determine the methylation status of *NKX2-5*_P in 2 TOF patients and in 1 control and BSP was used to measure the methylation level of *GATA4*_B in 2 TOF patients and in 2 controls to confirm the accuracy of the MALDI-TOF MassARRAY (Sequenom) methylation analysis. *NKX2-5*_P and *GATA4*_B overlapped with *NKX2-5*_M2 and *GATA4*_M (Figures 4A and 5A, respectively). Primer sequences used are listed in Additional file 1: Table S4.

As shown in Figure 4, the methylation value for *NKX2-5*_P was less than 10%. This result was similar to that found using the MassARRAY methylation analysis for *NKX2-5*_M2. Methylation analysis results from BSP for *GATA4*_B were also consistent with the MassARRAY methylation analysis results for *GATA4*_M (Figure 5). These data confirmed the accuracy of methylation analysis results from the MALDI-TOF MassARRAY (Sequenom).

### Validation of different methylation levels for *NKX2-5*_M3 and *HAND1*_M1

**Figure 6:**
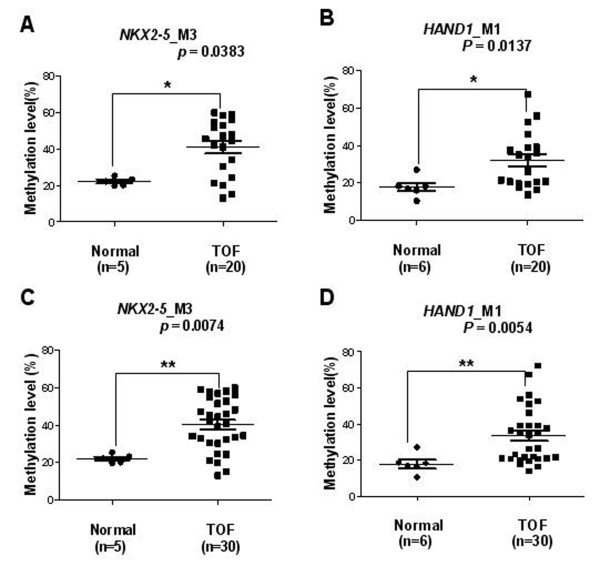
**Comparison of median methylation level for *NKX2-5*_M3 and *HAND1*_M1 in the control and TOF subjects. (A)** Median methylation level for *NKX2-5*_M3 of 5 control and 20 TOF subjects; **(B)** Median methylation level for *HAND1*_M1 of 6 control and 20 TOF subjects; **(C)** Median methylation level for *NKX2-5*_M3 of 5 control and 30 TOF subjects; **(D)** Median methylation level for *HAND1*_M1 of 6 control and 30 TOF subjects. *p <0.05, **p < 0.01, ***p < 0.001 (Mann–Whitney test).

The Sequenom MassARRAY platform was used to validate and compare the different methylation levels of *NKX2-5_*M3 and *HAND1*_M1 in 20 patients with TOF and 6 age matched controls. As shown in Figure 6A, the methylation level of *NKX2-5_*M3 was significantly higher in patients with TOF, with a median value of 45.06% (IQR: 25.93%–54.36%, N = 20) compared to 22.18% (IQR: 20.07%–24.31%, *p* = 0.0383,N = 5, one male, aged 1.9 years, not included) in controls. Furthermore, patients with TOF also had significantly higher methylation levels for *HAND1*_M1, with a median value of 30.01% (IQR: 19.82%–39.11%, N = 20) compared to 17.54% (IQR: 14.69%–20.64%; N = 6, *p* = 0.0137, Figure 6B) in controls.

The *NKX2-5_*M3 and *HAND1_*M1 methylation levels in the 20 TOF patients and the initial 10 TOF subjects were combined and compared to those of the control subjects. A significantly higher *NKX2-5*_M3 methylation level was found in TOF patients, with a median value of 41.65% (IQR: 30.46%–53.35%, N = 30) compared to 22.18% (IQR: 20.07%–24.31%, N = 5) in controls (*p* = 0.0074, Figure 6C). The *HAND1_*M1 methylation level was also significantly higher in TOF patients, with a median value of 30.05% (IQR: 20.77%–40.89%, N = 30) compared to 17.54% (IQR: 14.69%–20.64%, N = 6) in controls (*p* = 0.0054, Figure 6D).

### Expression levels of *NKX2-5* and *HAND1* mRNA in patients with TOF and controls

The mRNA expression levels of the *NKX2-5* and *HAND1* genes were determined by QRT-PCR in 30 TOF patients and 6 controls. The normalized Ct mean (ΔCt), ΔΔCt and RQ (2^-ΔΔCt^) values for *NKX2-5* and *HAND1* genes in each samples are listed in Additional file 1: Table S5. The RQ value (2^-ΔΔCt^) for control 1 was set as 1 and used for normalization for all samples.

As shown in Table 1, *NKX2-5* and *HAND1* mRNA relative expression levels (RQ values) were significantly lower in patients with TOF compared with controls (*p* < 0.05).

**Table 1:** **Relative mRNA expression levels of *NKX2-5 *** **and *HAND1 *** **in samples from controls and patients with TOF**

**Gene**	**Control (RQ value (2**^**-ΔΔCt**^**) **^**a**^**: mean ± SD,n = 6)**	**TOF (RQ value (2**^**-ΔΔCt**^**): mean ± SD, n = 30)**	***P *** **value**^**b**^
***NKX2-5***	9.76 ± 7.39^c^	1.48 ± 3.45	0.0028
***HAND1***	7.55 ± 5.62	1.20 ± 0.41	0.0032

^a^ The *RQ* values (2^-ΔΔCt^) for control 1 was set as 1 and used for normalization for all samples; ^b^ Mann–Whitney test was performed; ^c^ “±” represents the meaning of addition and subtraction. One male, aged 1.9 years, was not included.

### Association between the methylation statuses and mRNA levels of *NKX2-5* and *HAND1*

A correlation analysis was done, in TOF patients, to identify any relationship between *NKX2-5* and *HAND1* methylation status and their respective mRNA levels.

**Figure 7:**
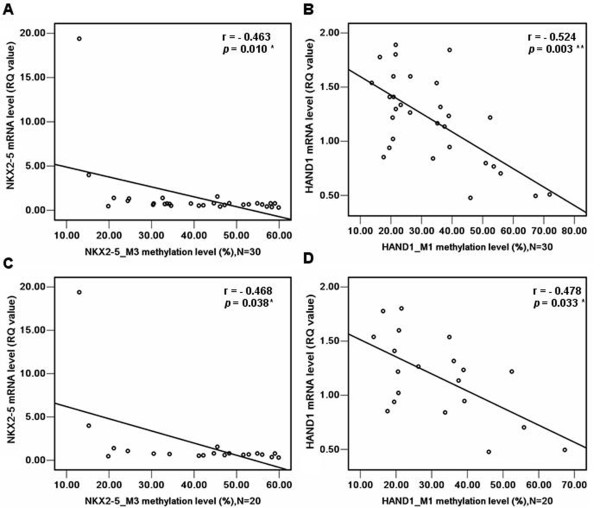
**Association of methylation status and mRNA levels for *NKX2-5*_M3 and *HAND1*_M1 in patients with TOF. (A)** Association of methylation status and mRNA levels for *NKX2-5*_M3 in 30 patients with TOF; **(B)** Association of methylation status and mRNA levels for *HAND1*_M1 in 30 patients with TOF; **(C)** Association of methylation status and mRNA levels for *NKX2-5*_M3 in 20 patients with TOF; **(D)** Association of methylation status and mRNA levels for *HAND1*_M1 in 20 patients with TOF. RQ values represent relative mRNA expression levels for *NKX2-5* and *HAND1* genes; the RQ value (2^-ΔΔCt^) for control 1 was set as 1 and used for normalization for all samples. *p < 0.05, **p < 0.01, ***p < 0.001 (Spearman).

As shown in Figure 7A, a significant association was observed between the methylation status and mRNA level of *NKX2-5*_M3 (r = -0.463, *p* = 0.010, N = 30). Similarly, there was a significant association between the methylation status and mRNA level of the *HAND1* gene (r = -0.524, *p* = 0.003, N = 30, Figure. 7B).

The association between *NKX2-5* and *HAND1* methylation status and their respective mRNA levels was also analyzed without the initial 10 TOF subjects. Interestingly, there was a significant correlation found between the *NKX2-5*_M3 methylation status and mRNA level (r = -0.468, *p* = 0.038, N = 20, Figure 7C). A significant association was also observed between the methylation status and mRNA level of *HAND1* (r = -0.478, *p* = 0.033, N = 20, Figure 7D).

## Discussion

Epigenetic control mechanisms play key roles in the regulation of tissue homeostasis and disease development. This includes DNA methylation, histone modification, the regulation of mRNA stability, translation by non-coding RNAs and differential RNA splicing [30]. DNA methylation constitutes an important epigenetic regulation mechanism in many eukaryotes and has been extensively studied [31]. In a previous study, decreased LINE-1 methylation levels were found in the cardiac tissue of TOF patients. The lower LINE-1 methylation level may be associated with increased TOF risk [27] and could serve as an indicator of global DNA methylation [32]. In the current study, we initially performed quantitative methylation analysis of *NKX2-5, GATA4* and *HAND1* genes in the right ventricular myocardium tissues of 10 TOF patients and 6 age matched control subjects using the Sequenom MassARRAY platform and found a significant difference in the methylation levels of *NKX2-5_*M3 and *HAND1*_M1 in TOF patients compared with controls (*p* < 0.05). The methylation values for *NKX2-5_*M3 and *HAND1*_M1 were then validated in a larger cohort TOF subjects. The difference in methylation levels for *NKX2-5*_M3 and *HAND1*_M1 were compared, between TOF patients and controls, in two independent cohorts. A significant difference was observed in the methylation status of *NKX2-5_*M3 when comparing the 20 TOF patients to the 6 controls (*p* = 0.0383). The methylation levels for the promoter region of the *HAND1* gene (*HAND1*_M1) were also significantly different between the 20 TOF patients and the 6 controls (*p* = 0.0137). Interestingly, the significant difference in the methylation levels for *NKX2-5_*M3 *and HAND1*_M1 increased when comparing the 30 TOF cases to the 6 controls (*p* = 0.0074, *p* = 0.0054, respectively).

The MassARRAY EpiTYPER^®^ for quantitative analysis of DNA methylation combines base-specific enzymatic cleavage with MALDI-TOF mass spectrometry. This combination provides a highly accurate, sensitive and high-throughput method for the quantitative analysis of DNA methylation. The EpiTYPER software provides convenient solutions for data analysis and export, however,the Sequenom EpiTYPER has a 5% technical error rate [33]. Although the robustness of this approach for confirming the accuracy of Sequenom methylation analysis results has been demonstrated, we used two alternate methods,pyrosequence and BSP,to test the methylation analysis results of *NKX2-5* and *GATA4*. No apparent differences were detected among these methods. This data supports the accuracy of the Sequenom MassARRAY methylation analysis results in our study.

Increasing evidence suggests that single gene mutations are present in a broad spectrum of genes involved in cardiac structure and function. Factors, such as *NKX2-5, GATA4* and *HAND1,* are among the earliest transcription factors expressed in the developing heart and are crucial in the activation of cardiac-specific genes [34]. Wang et al. sequenced all exons and their boundaries in the *NKX2-5, GATA4* and *HAND1* genes from non-syndromic TOF children and controls and found no evidence that somatic mutations in *NKX2-5, GATA4* and *HAND1* play a role in the pathogenesis of TOF [35]. Although mutations in *NKX*2-5, *GATA4* and *HAND*1 have been observed in patients with TOF, they are present only in a small percentage of TOF cases and cannot be considered to be the main cause of TOF. Based on these findings, we hypothesized that the aberrant DNA methylation modifies these genes and likely contributes to the development of TOF. In the present study, no significant difference in *GATA4* methylation levels were observed between TOF patients and controls, thus the methylation status of *GATA4* likely has no influence on its gene expression. Furthermore, we found that mRNA levels of the *NKX2-5* and *HAND1* genes were significantly lower in TOF patients compared to controls (*p* < 0.05). We also found significant negative correlations between methylation status and mRNA level for *NKX2-5* and *HAND1* in the 30 TOF cases (Figure 7A and B). Interestingly, the associations between methylation status and mRNA level for *NKX2-5* and *HAND1* were confirmed in the 20 TOF cases without including the initial 10 TOF subjects (Figure 7C and D). This demonstrates a consistent, significant association via two independent comparisons. These findings indicate that changes in the methylation levels for *NKX2-5* and *HAND1* may influence gene expression and contribute to the development of TOF.

One limitation of this study was that we were unable to obtain enough complete age-matched samples from TOF patients and healthy controls due to the difficulty of obtaining cardiac tissue samples. Further studies with larger number of subjects or samples are warranted in order to confirm our findings that aberrant methylation contributes to TOF development. Moreover, based only on the current results from clinical samples, we cannot ascertain whether the noted methylation changes occurred after the heart was already formed or after the heart defect already existed. Thus, we cannot determine whether these changes are reflective of disease physiology or related to disease etiology. Related research will be conducted using cell lines or animal models in future studies.

## Conclusion

In the present study, we examined the methylation status of *NKX2-5, GATA4* and *HAND1* and found significant changes in the methylation levels in the *NKX2-5* gene body and the *HAND1* gene promoter region. In addition, the aberrant methylation status of *NKX2-5* and *HAND1* was negatively correlated with their corresponding mRNA expression, indicating that the DNA methylation changes in these two genes contribute to transcription regulation in TOF patients and may play important roles in the pathogenesis of TOF. These findings may provide important clues for the development of novel treatments for TOF and provide a more thorough understanding of the etiology of congenital heart disease.

## Competing interests

The authors declare that they have no competing interests.

## Authors’ contributions

SW participated in study concept and design and coordination of the study, helped with the statistical analysis and drafted the manuscript. QY, WH, MX, DL and AQ participated in TOF sample acquisition and helped to draft the manuscript. ZP and CL participated in normal control sample acquisition and helped to draft the manuscript. MD and HG conceived of the study, and participated in its design and coordination and helped to draft the manuscript. All authors read and approved the final manuscript.

## Pre-publication history

The pre-publication history for this paper can be accessed here:

http://www.biomedcentral.com/1755-8794/6/46/prepub

## Supplementary Material

Additional file 1: Table S1 Anagraphical characteristics of study subjects. **Table S2.** Primer sequences, position, product length, and CpG unit used for MassArray quantitative methylation analysis. **Table S3.** Primer sequences and product length for QPT-PCR analysis. **Table S4.** Primer sets for pyrosequencing and bisulfite sequencing PCR (BSP). **Table S5.** The values of ΔCt mean,ΔΔCt and RQ for *NKX2-5* and *HAND1* in controls and TOF subjects. Click here for file
