# Supplementary material for: DNA methylation status of NKX2-5, GATA4 and HAND1 in patients with tetralogy of fallot
Source: BMC Med Genomics. 2013 Nov 1;6:46. doi: 10.1186/1755-8794-6-46 (PMC3819647; doi:10.1186/1755-8794-6-46)
Supplement: Additional file 1: Table S1 — Anagraphical characteristics of study subjects. Table S2. Primer sequences, position, product length, and CpG unit used for MassArray quantitative methylation analysis. Table S3. Primer sequences and product length for QPT-PCR analysis. Table S4. Primer sets for pyrosequencing and bisulfite sequencing PCR (BSP). Table S5. The values of ΔCt mean,ΔΔCt and RQ for NKX2-5 and HAND1 in controls and TOF subjects. [file 1755-8794-6-46-S1.doc]

**Additional file 1**

**Table S1．Anagraphical characteristics of study subjects**

| Characteristic | TOF^1^ , N =30 | Control , N=6 |
| --- | --- | --- |
| Age（mean ± SD, years）  Gender  Male (%)  Female (%) | 1.13 ± 0.85  20 (66.7 )  10 (33.3) | 1.73 ± 1.44  4 (66.7)  2 (33.3 ) |

^1^TOF, tetralogy of Fallot.

**Table S2. Primer sequences, position, product length, and CpG unit used for MassArray quantitative methylation analysis**

| Genes | Forward primer(5′ →3 ′)^1^ | Reverse primer (5′ →3 ′)^2^ | Position（bp） | Product  Length(bp) | CpG unit |
| --- | --- | --- | --- | --- | --- |
| *NKX2-5_*M1 | TTTTAGGTTTTGGGAGTGTGG | AAACCAAAAACTACCTAAAAACACA | -1596 ~ -1374 | 223 | 11 |
| *NKX2-5*_M2 | GAGGTTTGATTTTGTATGTTTTTGG | ACCCCTAACAACTTCCCTACATAAT | -159 ~ 217 | 377 | 17 |
| *NKX2-5*_M3 | AGGAGGGTTTTGGATTTTTTTT | ATTTATTCCCAAACCTCTACTCCTC | 1058 ~ 1524 | 467 | 25 |
| *GATA4*_M | GAGAGTTGAGTTTAAGAGGTTATTTTTTT | CCCTACCTACTAAACCTAAAAATTCC | -392 ~ 107 | 500 | 38 |
| *HAND1*_M1 | GAGGAGATTTGTTGGTTAGATGTTT | AATAAAAATTCCAACAATTCCCAAT | -887 ~ -414 | 474 | 30 |
| *HAND1*_M2 | TGGGAATTGTTGGAATTTTTATTTA | ACCCAAAAACCTATTTAACCCTTCTA | -436~ 62 | 499 | 20 |
| *HAND1*_M3 | TAGAAGGGTTAAATAGGTTTTTGGGT | TCAACAACCAACTCTAAAAATAAAACC | 37~ 398 | 362 | 22 |

^1^10-mer tag: cagtaatacgactcactatagggagaagg and ^2^T7 promoter tag: aggaagagag were added.

**Table S3． Primer sequences and product length for QPT-PCR analysis**

| Genes | Forward primer(5′ →3 ′) | Reverse primer(5′ →3 ′) | Product  length(bp) |
| --- | --- | --- | --- |
| ***NKX2-5*** | GGGCCTCAATCCCTACGGTTA | CCGAAGTTCACGAAGTTGTTG | 159 |
| ***HAND1*** | AGCCACCAGCTACATCGCCTAC | GCCATCCGCCTTCTTGAGTTC | 100 |
| ***B2M*** | TGCTGTCTCCATGTTTGATGTATCT | TCTCTGCTCCCCACCTCTAAGT | 161 |
| ***GAPDH*** | AGAAGGCTGGGGCTCATTTG | AGGGGCCATCCACAGTCTTC | 220 |

**Table S4． Primer sets for pyrosequencing and bisulfite sequencing PCR (BSP)**

| Primers for pyrosequence | Sequence | Position（bp） |
| --- | --- | --- |
| *NKX2-5*_M2 | Forward primer: TTTGGTTTAATGGTAGGTTGAGTTTT  Reverse primer: Biotin-CCCCTAACAACTTCCCTACATA  Sequencing primer: ATTTGTTGTTAGGATATATTTAGAG | 165 – 195 |
| Primers for BSP: |  |  |
| *GATA4*_M | Forward primer: AGAAAGGGAATTTATTAATAAAGTTGA  Reverse primer : ACCCTACCTACTAAACCTAAAAATTC | -296 – 116 |

**Table S5． The values of ΔCt mean，ΔΔCt and RQ for *NKX2-5* and *HAND1* in controls and TOF subjects**

| Sample ID | ***NKX2-5*** | | | ***HAND1*** | | |
| --- | --- | --- | --- | --- | --- | --- |
|  | **ΔCt Mean** | **ΔΔCt** | **RQ(2^-ΔΔCt^)^1^** | **ΔCt Mean** | **ΔΔCt** | **RQ(2^-ΔΔCt^)** |
| Control 1 | **4.972776** | **0** | **1.000000** | **6.445937** | **0** | **1.000000** |
| Control 2 | **2.058096** | **-2.91468** | **7.540613** | **3.745477** | **-2.70046** | **6.500112** |
| Control 3 | **0.743026** | **-4.22975** | **18.762120** | **3.064827** | **-3.38111** | **10.418760** |
| Control 4 | **2.495696** | **-2.47708** | **5.567704** | **2.351947** | **-4.09399** | **17.077110** |
| Control 5 | **0.978496** | **-3.99428** | **15.936660** | **3.760137** | **-2.6858** | **6.434357** |
| Control 6 |  |  |  | **4.488067** | **-1.95787** | **3.884893** |
|  |  |  |  |  |  |  |
| TOF patient 1 | **5.912121** | **0.939345** | **0.5214695** | **6.069667** | **-0.37627** | **1.297986** |
| TOF patient 2 | **5.648135** | **0.675359** | **0.6261762** | **5.950397** | **-0.49554** | **1.409850** |
| TOF patient 3 | **4.569446** | **-0.40333** | **1.322560** | **7.420135** | **0.974198** | **0.5090228** |
| TOF patient 4 | **5.476834** | **0.504058** | **0.7051204** | **5.563597** | **-0.88234** | **1.843369** |
| TOF patient 5 | **6.209777** | **1.237001** | **0.4242536** | **6.028127** | **-0.41781** | **1.335898** |
| TOF patient 6 | **6.209777** | **1.237001** | **0.4242536** | **6.224347** | **-0.22159** | **1.166021** |
| TOF patient 7 | **5.334553** | **0.361777** | **0.7782056** | **5.527017** | **-0.91892** | **1.890704** |
| TOF patient 8 | **5.366331** | **0.393555** | **0.7612516** | **6.827805** | **0.381868** | **0.7674432** |
| TOF patient 9 | **4.497566** | **-0.47521** | **1.390119** | **5.768417** | **-0.67752** | **1.599387** |
| TOF patient 10 | **5.366331** | **0.393555** | **0.7612516** | **6.771287** | **0.32535** | **0.7981045** |
| TOF patient 11 | **4.339436** | **-0.63334** | **1.551151** | **5.768417** | **-0.67752** | **1.599387** |
| TOF patient 12 | **5.712285** | **0.739509** | **0.5989432** | **5.950397** | **-0.49554** | **1.409850** |
| TOF patient 13 | **6.650402** | **1.677626** | **0.3125967** | **7.459259** | **1.013322** | **0.4954041** |
| TOF patient 14 | **5.461144** | **0.488368** | **0.712831** | **6.048947** | **-0.39699** | **1.316757** |
| TOF patient 15 | **4.497566** | **-0.47521** | **1.390119** | **6.415167** | **-0.03077** | **1.021559** |
| TOF patient 16 | **4.876406** | **-0.09637** | **1.069083** | **5.825167** | **-0.62077** | **1.537699** |
| TOF patient 17 | **0.695126** | **-4.27765** | **19.395500** | **6.142317** | **-0.30362** | **1.234234** |
| TOF patient 18 | **6.077684** | **1.104908** | **0.464932** | **6.95382** | **0.507883** | **0.7032534** |
| TOF patient 19 | **5.798397** | **0.825621** | **0.5642391** | **6.159927** | **-0.28601** | **1.219260** |
| TOF patient 20 | **6.428847** | **1.456071** | **0.3644843** | **5.616027** | **-0.82991** | **1.777580** |
| TOF patient 21 | **5.322925** | **0.350149** | **0.7845033** | **6.674335** | **0.228398** | **0.8535822** |
| TOF patient 22 | **5.590002** | **0.617226** | **0.6519234** | **5.596267** | **-0.84967** | **1.802084** |
| TOF patient 23 | **2.975676** | **-1.9971** | **3.991979** | **7.512996** | **1.067059** | **0.4772911** |
| TOF patient 24 | **5.648135** | **0.675359** | **0.6261762** | **6.106627** | **-0.33931** | **1.265153** |
| TOF patient 25 | **5.912121** | **0.939345** | **0.5214695** | **6.695326** | **0.249389** | **0.8412528** |
| TOF patient 26 | **5.334553** | **0.361777** | **0.7782056** | **6.524431** | **0.078494** | **0.947046** |
| TOF patient 27 | **5.362518** | **0.389742** | **0.7632662** | **6.536326** | **0.090389** | **0.9392692** |
| TOF patient 28 | **5.308883** | **0.336107** | **0.7921761** | **6.261947** | **-0.18399** | **1.136025** |
| TOF patient 29 | **5.547851** | **0.575075** | **0.6712513** | **5.823867** | **-0.62207** | **1.539083** |
| TOF patient 30 | **5.303019** | **0.330243** | **0.7954023** | **6.160727** | **-0.28521** | **1.218584** |

**^1^ RQ(2^-ΔΔCt^), Relative Quantification.**
